# Supplementary material for: Impact of Nutritional Status on Severe Radiation-Induced Mucositis in Oropharyngeal Cancer Patients Undergoing Chemo-Radiotherapy
Source: Nutrients. 2025 Oct 21;17(20):3301. doi: 10.3390/nu17203301 (PMC12566891; doi:10.3390/nu17203301)
Supplement: Supplementary file 1 [file nutrients-17-03301-s001.zip › nutrients-3912900-supplementary.pdf]

## Supplementary Table S1. Socioeconomic characteristics of patients.

| | Variable | Category | N= 82 |
|---|---|---|---|
| Social status | Married | 51 | (62.2) |
| | Single | 15 | (18.3) |
| | Separated | 11 | (13.4) |
| | Other | 5 | (6.1) |
| Home support | Alone | 18 | (22) |
| | Not alone | 64 | (78) |
| Employment status | Working | 28 | (34.1) |
| | Retired | 35 | (42.7) |
| | Other | 19 | (23.2) |

## Supplementary Figure S1. ROC curves stratified by sex.

A. Men

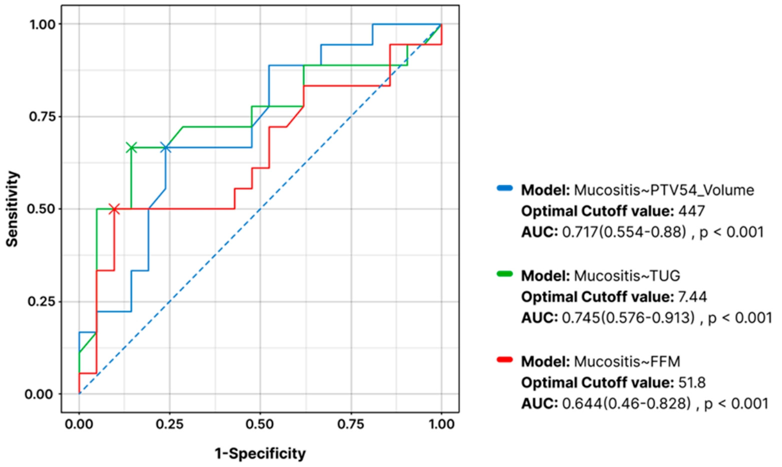


B. Women

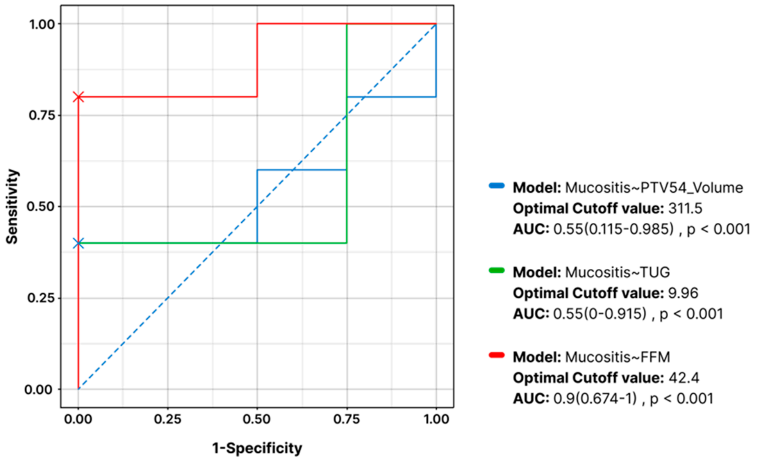


ROC curve analysis of PTV45 volume, TUG and FFM to assess severe radiation-induced mucositis according to Grade 3 CTCAE V5.0 in oropharyngeal cancer patients undergoing chemo-radiotherapy. **(a)** Men. **(b)** Women. ROC: Receiver operating characteristic. PTV54: Planning Tumor Volume at a prescribed dose of 54Gy. FFM: Fat-Free Mass. TUG: Timed Up and Go. PPV: Positive Predictive Value. NPV: Negative Predictive Value. AUC: Area Under the Curve. Sensitivity and specificity data: For FFM: Sensitivity 61.9%. Specificity 80%. PPV 72.2%. NPV 71.4%. Optimal cutoff value 51.8Kg (AUC = 0.692; 95% CI: 0.534–0.851; $p = 0.001$. For PTV54: Sensitivity 52.4%. Specificity 80%. PPV 68.8%. NPV 66.7%. Optimal cutoff value $455m^3$ (AUC = 0.66; 95% CI: 0.498–0.822; $p = 0.001$. For TUG: Sensitivity 66.7%. Specificity 84%. PPV 77.8%. NPV 75%. Optimal cutoff value 7.44 seconds (AUC = 0.763; 95% CI: 0.619–0.906; $p = 0.001$

## Supplementary Table S2. Correlation heatmap with p values.

| | | | | | | | |
|---|---|---|---|---|---|---|---|
| GLIM | | | | | | | 1 |
| MUCOSITIS | | | | | | 1 | **0,32 p=0,026** |
| TUG | | | | | 1 | 0,25 p=0,09 | 0,08 p=0,57 |
| DINMAX | | | | 1 | -0,26 p=0,07 | -0,13 p=0,38 | **-0,42 p=0,003** |
| RF-CSA | | | 1 | **0,47 p=0,001** | -0,21 p=0,16 | -0,21 p=0,15 | **-0,34 p=0,02** |
| BCM | | 1 | **0,75 p=0,001** | **0,53 p=0,001** | -0,14 p=0,35 | -0,19 p=0,19 | **-0,4 p=0,006** |
| FFM | 1 | **0,83 p=0,001** | **0,58 p=0,001** | **0,58 p=0,001** | 0,03 p=0,79 | **-0,32 p=0,03** | **-0,43 p=0,003** |
| | FFM | BCM | RF-CSA | DINMAX | TUG | MUCOSITIS | GLIM |

Correlation heatmap of BIVA variables, ultrasound variables, functional test, radiation-induced mucositis grade and nutritional status in head and neck cancer patients undergoing chemoradiotherapy. **Bold** indicates significant p-values. BIVA: Bioelectrical Impedance Vector Analysis. FFM: Fat-Free Mass. BCM: Body Cell Mass. RF-CSA: Rectus femoris cross-sectional area. TUG: Timed Up and Go. GLIM: Global Leadership Initiative on Malnutrition.

## Supplementary Table S3. Odds ratio table (multivariate analysis). Model 3.

| Variable | p value | OR | CI 95% |
|---|---|---|---|
| Age | 0.754 | 1.01 | 0.94 – 1.09 |
| BMI | 0.723 | 1.02 | 0.90 – 1.16 |
| PTV54 | 0.076 | 1.00 | 1.00 – 1.01 |
| GLIM | 0.011 | 19.25 | 2.84 – 404.82 |

**Model 3**: Predictive additional factors for severe oral radiation-induced mucositis including BMI. OR: Odds Ratio. CI: Confidence Interval. BMI: Body Mass Index. PTV54: Planning Tumor Volume at a prescribed dose of 54Gy. GLIM: Global Leadership Initiative on Malnutrition.
